# New lineages of RNA viruses from clinical isolates of *Rhizopus microsporus* revealed by fragmented and primer-ligated dsRNA sequencing (FLDS) analysis

**DOI:** 10.1128/msphere.00345-24

**Published:** 2024-07-29

**Authors:** Wasiatus Sa'diyah, Yan-Jie Zhao, Yuto Chiba, Hideki Kondo, Nobuhiro Suzuki, Sayaka Ban, Takashi Yaguchi, Syun-ichi Urayama, Daisuke Hagiwara

**Affiliations:** 1 Department of Life and Environmental Sciences, Laboratory of Fungal Interaction and Molecular Biology (Donated by IFO), University of Tsukuba, Ibaraki, Japan; 2 Institute of Plant Science and Resources, Okayama University, Kurashiki, Okayama, Japan; 3 Medical Mycology Research Center, Chiba University, Chiba, Japan; 4 Microbiology Research Center for Sustainability (MiCS), University of Tsukuba, Tsukuba, Japan; Wake Forest University, Winston-Salem, North Carolina, USA

**Keywords:** *Rhizopus microsporus*, RNA virus, diversity, new lineage, FLDS

## Abstract

*Rhizopus microsporus* is a species in the order Mucorales that is known to cause mucormycosis, but it is poorly understood as a host of viruses. Here, we examined 25 clinical strains of *R. microsporus* for viral infection with a conventional double-stranded RNA (dsRNA) assay using agarose gel electrophoresis (AGE) and the recently established fragmented and primer-ligated dsRNA sequencing (FLDS) protocol. By AGE, five virus-infected strains were detected. Then, full-length genomic sequences of 12 novel RNA viruses were revealed by FLDS, which were related to the families *Mitoviridae, Narnaviridae,* and *Endornaviridae,* ill-defined groups of single-stranded RNA (ssRNA) viruses with similarity to the established families *Virgaviridae* and *Phasmaviridae,* and the proposed family “Ambiguiviridae.” All the characterized viruses, except a potential phasmavirid with a negative-sense RNA genome, had positive-sense RNA genomes. One virus belonged to a previously established species within the family *Mitoviridae*, whereas the other 11 viruses represented new species or even new genera. These results show that the fungal pathogen *R. microsporus* harbors diverse RNA viruses and extend our understanding of the diversity of RNA viruses in the fungal order Mucorales, division Mucoromycota. Identifying RNA viruses from clinical isolates of *R. microsporus* may expand the repertoire of natural therapeutic agents for mucormycosis in the future.

**IMPORTANCE:**

The diversity of mycoviruses in fungal hosts in the division Mucoromycota has been underestimated, mainly within the species *Rhizopus microsporus*. Only five positive-sense RNA genomes had previously been discovered in this species. Because current sequencing methods poorly complete the termini of genomes, we used fragmented and primer-ligated double-stranded RNA sequencing to acquire the full-length genomes. Eleven novel mycoviruses were detected in this study, including the first negative-sense RNA genome reported in *R. microsporus*. Our findings extend the understanding of the viral diversity in clinical strains of Mucoromycota, may provide insights into the pathogenesis and ecology of this fungus, and may offer therapeutic options.

## INTRODUCTION

Most fungus-infecting viruses, known as mycoviruses, have positive-sense (+) single-stranded RNA (ssRNA) genomes, negative-sense (−) ssRNA genomes, or double-stranded RNA (dsRNA) genomes, but few of them have DNA genomes. A large number of mycoviruses have already been reported, especially from phytopathogenic ascomycetes and basidiomycetes (1–6). For example, Cryphonectria hypovirus 1 has received much attention due to its successful use in biocontrol (7, 8). Virome studies have also investigated numerous mycoviruses from grapevine downy mildew (9), endomycorrhizal fungi (10), and *Xylariaceae* fungi infecting avocado (11), and surprisingly, the mycoviruses detected in these studies have revealed new lineages (9, 10). A relatively small number of viruses infecting other fungi pathogenic to humans (12, 13) or insects have also been reported ((14),(15)

*Rhizopus microsporus* is a member of the order Mucorales in the division Mucoromycota and is known to be the leading etiological agent of mucormycosis, causing severe symptoms and even death (16–18). This fungus is an airborne pathogen commonly distributed in environments throughout the world (19, 20). The earliest studied mycovirus in *R. microsporus* had a +ssRNA genome and belonged to the family *Narnaviridae* (21). That study reported two partial sequences of RNA viruses in a fungal–bacteria symbiosis (21). Later, three full-length sequences of mycoviruses were detected in clinical isolates of *R. microsporus*, which belonged to the families *Endornaviridae* and *Mitoviridae* (13). The five mycoviruses reported thus far have +ssRNA genomes. Further viral screening to identify novel viruses in *R. microsporus* should extend our understanding of the diversity of mycoviruses beyond those with +ssRNA genomes.

Several methods have been used to screen for mycoviruses. Agarose gel electrophoresis (AGE) of viral dsRNA, which could be the genomes of dsRNA viruses or the replicative intermediate forms of ssRNA viruses, is the most commonly used method of detecting mycoviruses with RNA genomes (22, 23). The insensitivity of this method sometimes causes RNA mycoviruses to be overlooked. The Illumina sequencing method has recently shown greater detection sensitivity than AGE in this context, and viruses not detected with AGE have been detected with RNA sequencing (RNA-seq) (24, 25). However, the terminal sequences of viral genomes or their segments are commonly lacking with the RNA-seq method. Therefore, the rapid amplification of cDNA ends (RACE) method is required to obtain the full-length sequence of each RNA viral segment (26, 25). To overcome these limitations in future studies, fragmented and primer-ligated dsRNA sequencing (FLDS) was developed in 2016 (27, 28). This method allows the detection of the full-length RNA genomes of even multisegmented RNA viruses, without a RACE assay (24, 27–32).

We were interested in investigating the diversity of mycoviruses from clinical strains of *R. microsporus* with different methods. Here, we report the detection of 12 mycoviruses: 10 novel +ssRNA viruses and 1 novel −ssRNA virus, together with one previously reported mitovirus. The novel viruses were classified in the families *Mitoviridae* and *Narnaviridae*, a possible new lineage in the family *Endornaviridae,* and ill-defined groups related to the established families *Virgaviridae* and *Phasmaviridae* and the proposed family “Ambiguiviridae.”

## RESULTS

### Screening of dsRNA carrying *R. microsporus* strains

**Fig 1:**
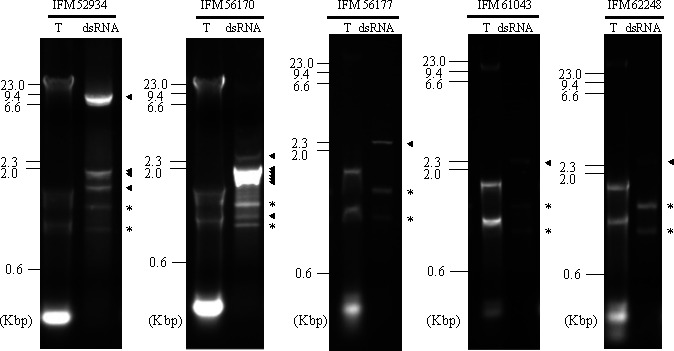
Agarose gel electrophoresis of total nucleotide (T) and dsRNA-enriched fractions (dsRNA) from dsRNA-positive clinical viral strains of *Rhizopus microsporus*. Relative positions of the DNA molecular weight markers are shown on the left of each panel. kbp, kilobase pairs. The bands presumably corresponding to the host RNA remnants are indicated by asterisk. The bands presumably corresponding to RNA virus are indicated by a black arrowhead.

Before dsRNA screening, the taxonomic positions of the 25 clinical fungal strains were confirmed, identifying them as *R. microsporus* with the universal fungal internal transcribed spacer (ITS) region, with a few nucleotides different (see Table S1). The dsRNA fractions extracted from these *R. microsporus* strains were then analyzed. AGE revealed that 5 (IFM 52934, IFM 56170, IFM 56177, IFM 61043, and IFM 62248) of the 25 strains (20%) of *R. microsporus* were positive for dsRNA, which was inferred to originate from RNA mycoviruses (Fig. 1). Multiple dsRNA bands with an estimated size of 1–9 kbp appeared in the nucleic acids extracted from all strains, but their patterns varied among the strains.

### Genome sequence determination of RNA viruses in *R. microsporus* strains

**Fig 2:**
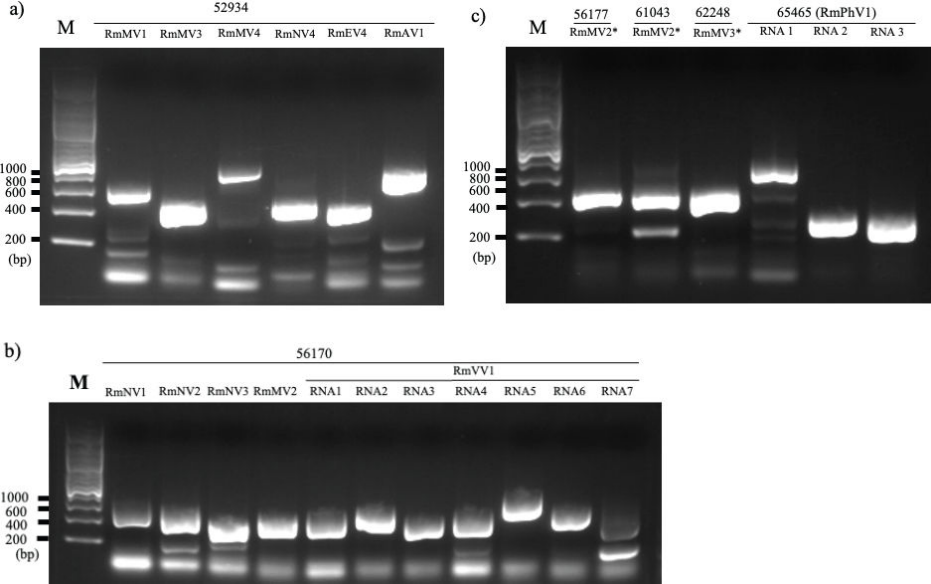
Detection of viruses from clinical strains of *Rhizopus microsporus* with RT-PCR. M, DNA marker ladder; *, variant.

To identify the *R. microsporus* viruses, some of which might be missed with dsRNA-based screening, we used FLDS to analyze all 25 *R*. *microsporus* strains, including the dsRNA-positive strains. From the sequence data of FLDS, 12 virus-like contigs containing an open reading frame (ORF) similar to that encoding RNA-dependent RNA polymerase (RdRP) were generated (see Materials and Methods; Table 1). These contigs were detected in six of the 25 *R*. *microsporus* strains with reverse transcription (RT)-PCR (Fig. 2). Five *R. microsporus* strains were dsRNA-positive, as mentioned above, whereas an additional viral sequence was detected in strain IFM 65465, which was negative on dsRNA screening (Table S1). Strains IFM 56170 and IFM 52934 were infected by multiple viruses, whereas the other four strains were infected by a single virus. The secondary structures of the viral untranslated regions were predicted (Fig. S2), to perceive those that may be involved in viral replication or may be the target of the exonuclease activity of the fungal host (33, 34).

**TABLE 1:** The detailed information of mycoviruses identified in *Rhizopus microsporus*

Host	Viral name (abbreviation)	RNA	Length	Gene	Blastp hit/percentage	Average coverage	Accession number
IFM 52934	*Rhizopus microsporus* mitovirus 1 (RmMV1)	RNA1	2,275	RdRP	*Rhizopus microsporus* mitovirus 1/97.03%	492.4659	LC818057
	*Rhizopus microsporus* mitovirus 3 (RmMV3)	RNA1	2,443	RdRP	*Mitovirus* sp./37.31%	11,074.75	LC818058
	*Rhizopus microsporus* mitovirus 4 (RmMV4)	RNA1	2,354	RdRP	*Entomophthora muscae* mitovirus 5/49,19%	6,993.604	LC818059
	*Rhizopus microsporus* narnavirus 4 (RmNV4)	RNA1	2,576	RdRP	Erysiphe necator-associated narnavirus 42/51.34%	146.7161	LC818060
	*Rhizopus microsporus* endornavirus 3 (RmEV3)	RNA1	9,286	RdRP	Almopos endorna-like virus 1/31.95%	1,6136.59	LC818061
	*Rhizopus microsporus* ambigui-like virus 1 (RmAV1)	RNA1	3,362	RdRP	*Riboviria* sp./42.23%	3,345.196	LC818062
IFM 56170	*Rhizopus microsporus* narnavirus 1 (RmNV1)	RNA1	2,874	RdRP	Putative Narnaviridae, partial/75.82%	1,124.573	LC818063
	*Rhizopus microsporus* narnavirus 2 (RmNV2)	RNA1	2,676	RdRP	*Rhizopus oryzae* narnavirus 1/57.85%	1,223.303	LC818064
	*Rhizopus microsporus* narnavirus 3 (RmNV3)	RNA1	2,546	RdRP	Sanya narnavirus 3/33.33%	668.5841	LC818065
	*Rhizopus microsporus* mitovirus 2 (RmMV2)	RNA1	2,441	RdRP	*Mitovirus* sp./37.92%	67,383.94	LC818066
	*Rhizopus microsporus* virga-like virus 1 (RmVV1)	RNA1	4,609	Met, Hel	*Bemisia tabaci* bromo-like virus 2/24.88%	762.8217	LC818067
		RNA2	4,593	RdRP	*Acidomyces richmondensis* tobamo-like virus 1/26.39%	92.82942	LC818068
		RNA3	3,808		*Aspergillus flavus* virga-like virus 1/26.21%	58.91159	LC818069
		RNA4	2,885			83.39827	LC818070
		RNA5	2,661			809.1535	LC818071
		RNA6	2,484		*Rhizopus arrhizus*/30.20%	227.4692	LC818072
		RNA7	1,525		*Mucor plumbeus*/32.49%	5,255.765	LC818073
IFM 56177	*Rhizopus microsporus* mitovirus 2 (RmMV2)	RNA1	2,441	RdRP	*Mitovirus* sp./37.77%	22,605.12	LC818074
IFM 61043	*Rhizopus microsporus* mitovirus 2 (RmMV2)	RNA1	2,439	RdRP	*Mitovirus* sp./38.23%	28,502.58	LC818075
IFM 62248	*Rhizopus microsporus* mitovirus 3 (RmMV3)	RNA1	2,445	RdRP	*Mitovirus* sp./37.88%	14,243.87	LC818076
IFM 65465	*Rhizopus microsporus* phasma-like virus 1 (RmPhV1)	RNA1	7,690	RdRP	*Suillus luteus*-associated bunya-like virus 2/69.24%	3,076.8	LC818077
		RNA2	798		*Mucor lusitanicus*/51.37%	9,951.8	LC818078
		RNA3	747		*Streptomyces* sp./54.79%; *Mortierella alpina*/51.58%	22,879.4	LC818079

### Novel +ssRNA mitoviruses from *R. microsporus*

**Fig 3:**
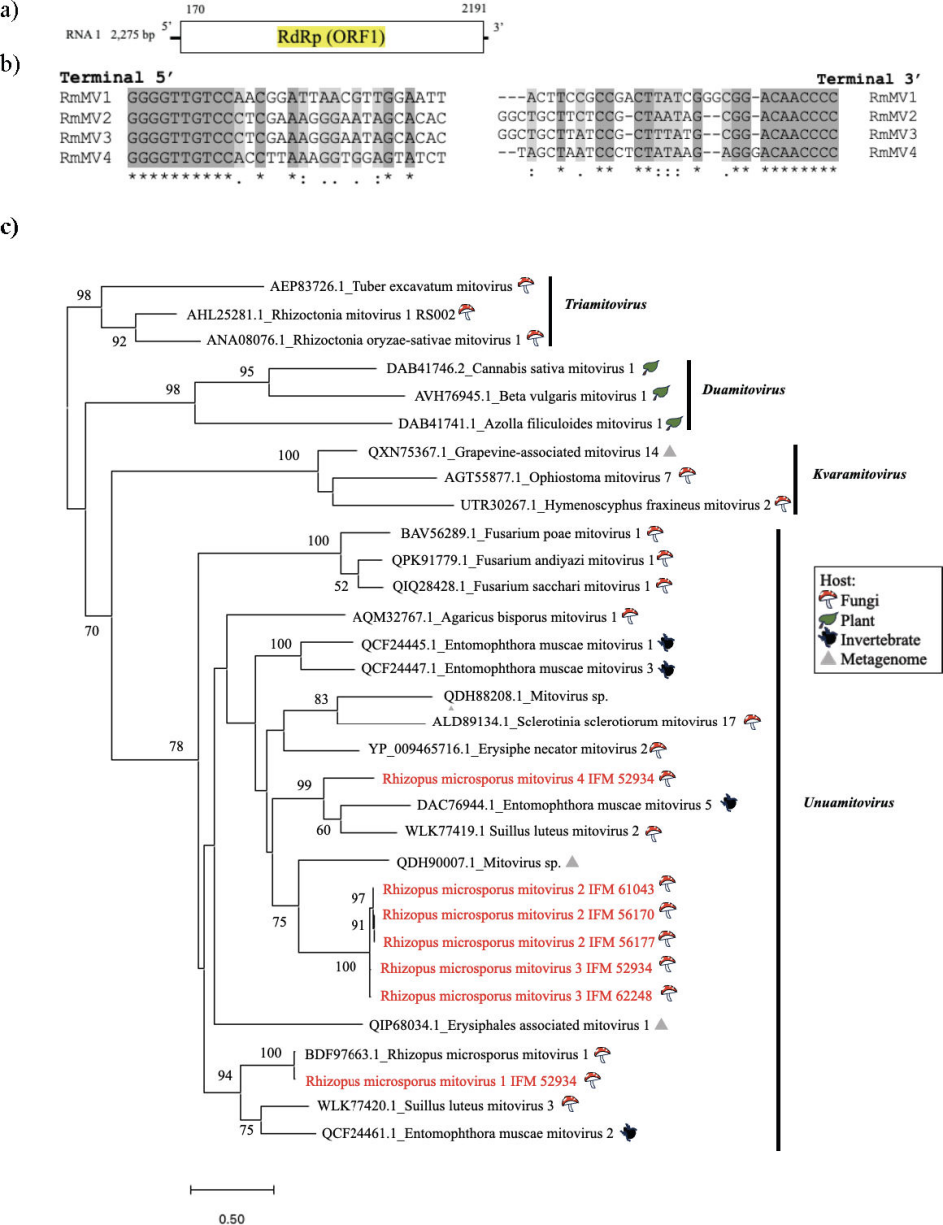
Characterization of mitoviruses from clinical strains of *R. microsporus*. (**a**) Genome organization of a representative *Rhizopus microsporus* mitovirus (RmMV1); predicted ORF is indicated by the white box. (**b**) Comparison of the 5′ and 3′ termini of the RmMV1–4 genomes (represented as DNA sequence). (**c**) Phylogenetic tree of RdRPs of RmMV1–4 variants and selected mitovirids computed with RAxML and the PROTGAMMALG model, with 1,000 bootstrap replicates. Red font indicates mycoviruses found in this study. The scale bar shows the number of substitutions per site.

Viruses in the family *Mitoviridae* have a single +ssRNA genome (2.2–3.6 kb) encoding RdRP. They are thought to replicate in the host mitochondria and to be dependent on the mitochondrial codons (35). In the present study, four mitovirus candidates (approximately 2.3–2.4 kb) were identified (Table 1). One, from strain IFM 52934, showed 96.8% similarity to the RdRP sequence of a known mitovirus, *Rhizopus microsporus* mitovirus 1 (RmMV1, LC671615), and was considered to be a strain of this virus (13). Three others showed no significant sequence similarity (<80% aa identity; see also below) to known mitovirid sequences and were, therefore, tentatively designated *Rhizopus microsporus* mitovirus 2–4 (RmMV2–4) (Table 1). RmMV2 was detected in strains IFM 56170, IFM 56177, and IFM 61043 and shared >95% amino acid and >90% nucleotide sequence identity. RmMV3 was detected in strains IFM 52934 and IFM 62248 and shared >95% amino acid and nucleotide sequence similarity. RmMV2 and RmMV3 shared ≤90% amino acid sequence identity. RmMV4 was detected in strain IFM 52934. Based on BLAST searches, the amino acid sequences of the RdRP proteins of RmMV2, 3, and 4 shared 37.92% similarity with *Mitovirus* sp. (QDH90007), 37.31% with *Mitovirus* sp. (QDH90007), and 49.19% with *Entomophthora muscae* mitovirus 5 (DAC76944.1). A phylogenetic analysis based on the amino acid sequences of their RdRPs suggested that the *R. microsporus* mitoviruses detected in this study were related to members of the genus *Unuamitovirus* in the family *Mitoviridae* (Fig. 3c). According to the International Committee on Taxonomy of Viruses species demarcation criteria for mitoviruses (≤90% amino acid sequence identity in RdRP; https://talk.ictvonline.org/), RmMV2, RmMV3, and RmMV4 can be considered members of a new species in the family *Mitoviridae*.

### Novel +ssRNA narnaviruses from *R. microsporus*

**Fig 4:**
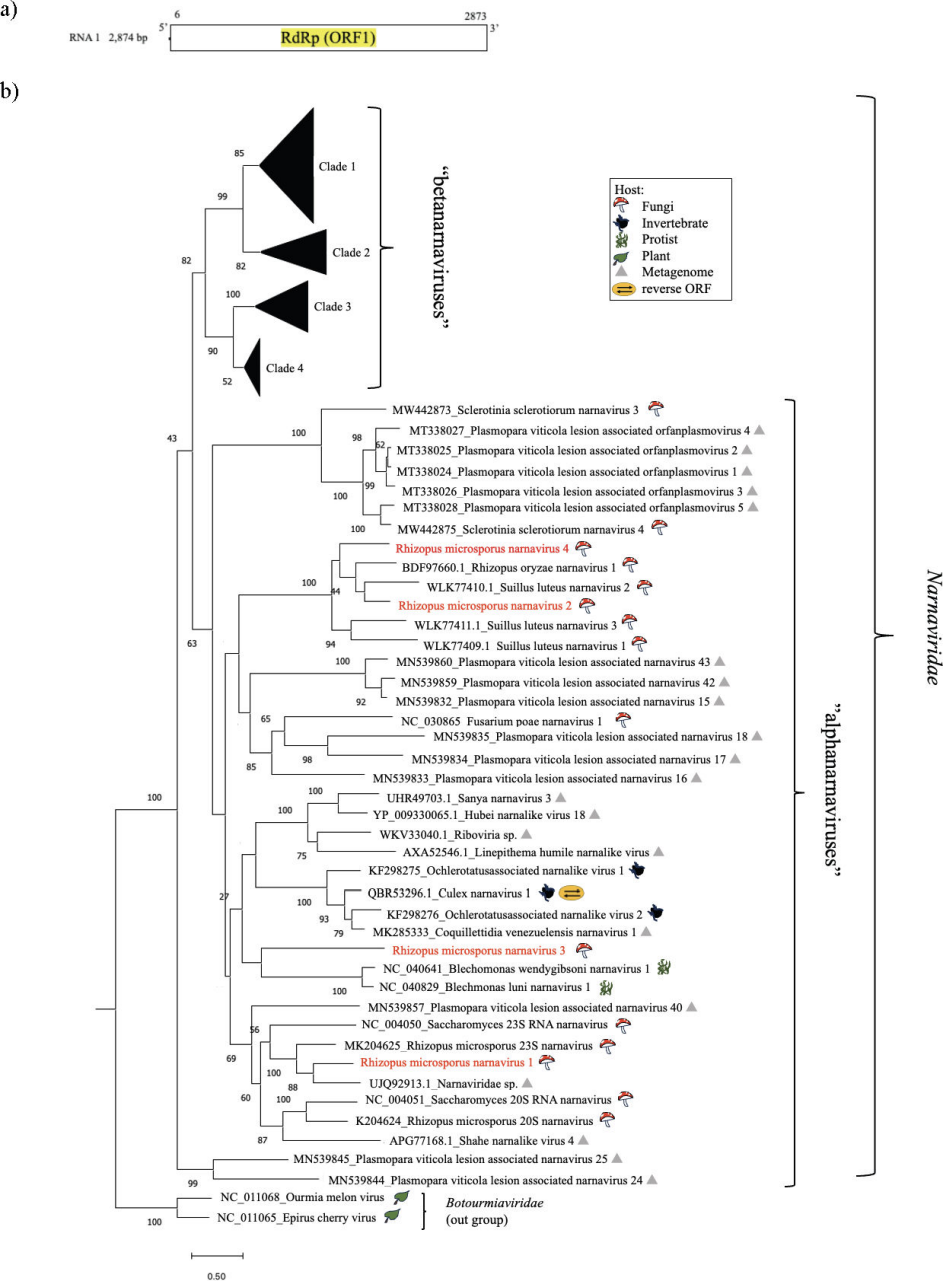
Characterization of narnaviruses from clinical strains of *R. microsporus.* (a) Genome organization of a representative *Rhizopus microsporus* narnavirus (RmNV1); predicted ORF is indicated by the white box. (**b**) Phylogenetic tree of RdRPs of RmNV1–4 computed with RAxML and the PROTGAMMALG model, with 1,000 bootstrap replicates. Red font indicates mycoviruses found in this study. The scale bar shows the number of substitutions per site. (See the supplemental material for *Narnaviridae sensu lato.*)

Members of the family *Narnaviridae* are regarded as nonencapsulated +ssRNA viruses and encode only RdRP (36, 37). Four narna-like virus strains were detected in *R. microsporus* strains IFM 56170 and IFM 52934 (Table 1). We tentatively designated them *Rhizopus microsporus* narnavirus 1–4 (RmNV1–4). The lengths of the RNA genomes of RmNV1–4 were 2.8, 2.6, 2.5, and 2.5, respectively (Fig. 4a). The genomic sequences of RmNV2 and 4 showed 74.63% similarity with one another. Based on a BLAST search of the RdRP amino acid sequence, RmNV1–4 showed 75.82% identity with *Narnaviridae* sp. (UJQ92731.1), 57.85% identity with *Rhizopus oryzae* narnavirus 1 (BDF97660.1), 33.33% identity with Sanya narnavirus 3 (UHR49703.1), and 51.34% identity with Erysiphe necator-associated narnavirus 42 (QJT93774.1). In a phylogenetic analysis of RdRP sequences, RmNV1–4 clustered in the proposed phylogroup “alphanarnavirus,” with classical members of *Narnaviridae*: *Saccharomyces* 23S RNA narnavirus and *Saccharomyces* 20S RNA narnavirus (Fig. 4b; Fig. S1). RmNV1 formed a clade with the *Rhizopus microsporus* 23S narnavirus from the previous study (Fig. 4b). However, the blast result showed only a 50% similarity of amino acid, and thus, RmNV1 is a novel virus in this study.

The classification of authentic narnaviruses has progressed, except for the two classical yeast viruses. RmNV1 belongs to the same clade with these yeast viruses, and it is reasonable to infer that it is a member of the genus *Narnavirus*.

### Novel +ssRNA endornavirus from *R. microsporus*

**Fig 5:**
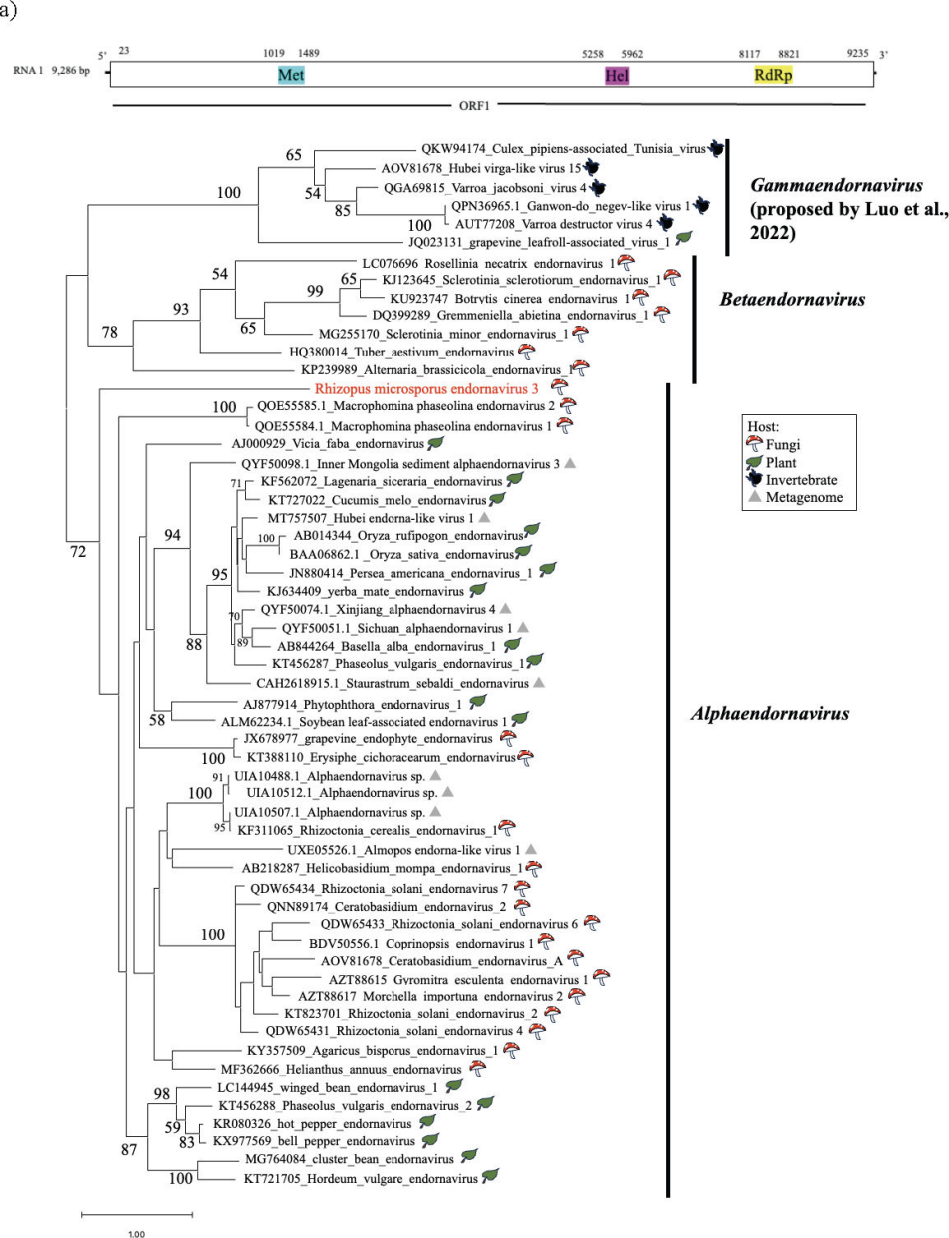
Characterization of RmEV3. (**a**) Genome organization of RmEV3; predicted ORF is indicated by the white boxes, with the three conserved domains (Met, Hel, and RdRP). (**b**) Phylogenetic tree of RdRPs of RmEV3 and other endornavirids computed with RAxML and the PROTGAMMALG model, with 1,000 bootstrap replicates. Red font indicates the mycoviruses found in this study. The scale bar shows the number of substitutions per site.

Viruses of the family *Endornaviridae* have a capsidless +ssRNA genome of 9.7–17.6 kb (38). In the present study, a candidate endornavirus was detected in strain IFM 52934 and designated *Rhizopus microsporus* endornavirus 3 (RmEV3). The complete genomic sequence of RmEV3 is 9.2 kb and consists of a single ORF encoding a methyl transferase, viral helicase superfamily 1, and RdRP (Fig. 5a). The RmEV3 genome is shorter than those of typical endornaviruses, including other *R. microsporus* endornaviruses, RmEV1 and RmEV2 (13). A BLASTx search revealed that the amino acid sequence of RdRP of this virus shared the greatest identity (31.95%) with Almopos endorna-like virus 1 (UXE05526.1). Therefore, the phylogenetic analysis based on amino acid sequences of RdRPs showed that RmEV3 is related to the genus *Alphaendornavirus* but distinct from the genus *Betaendornavirus* and the proposed genus “Gammaendornavirus” (39) (Fig. 5b). The phylogenetic analysis suggested that RmEV3 may be considered a novel member of the *Alphaendornavirus*, but further analysis of related viruses is required to confirm this proposition.

### Novel +ssRNA ambigui-like virus from *R. microsporus*

**Fig 6:**
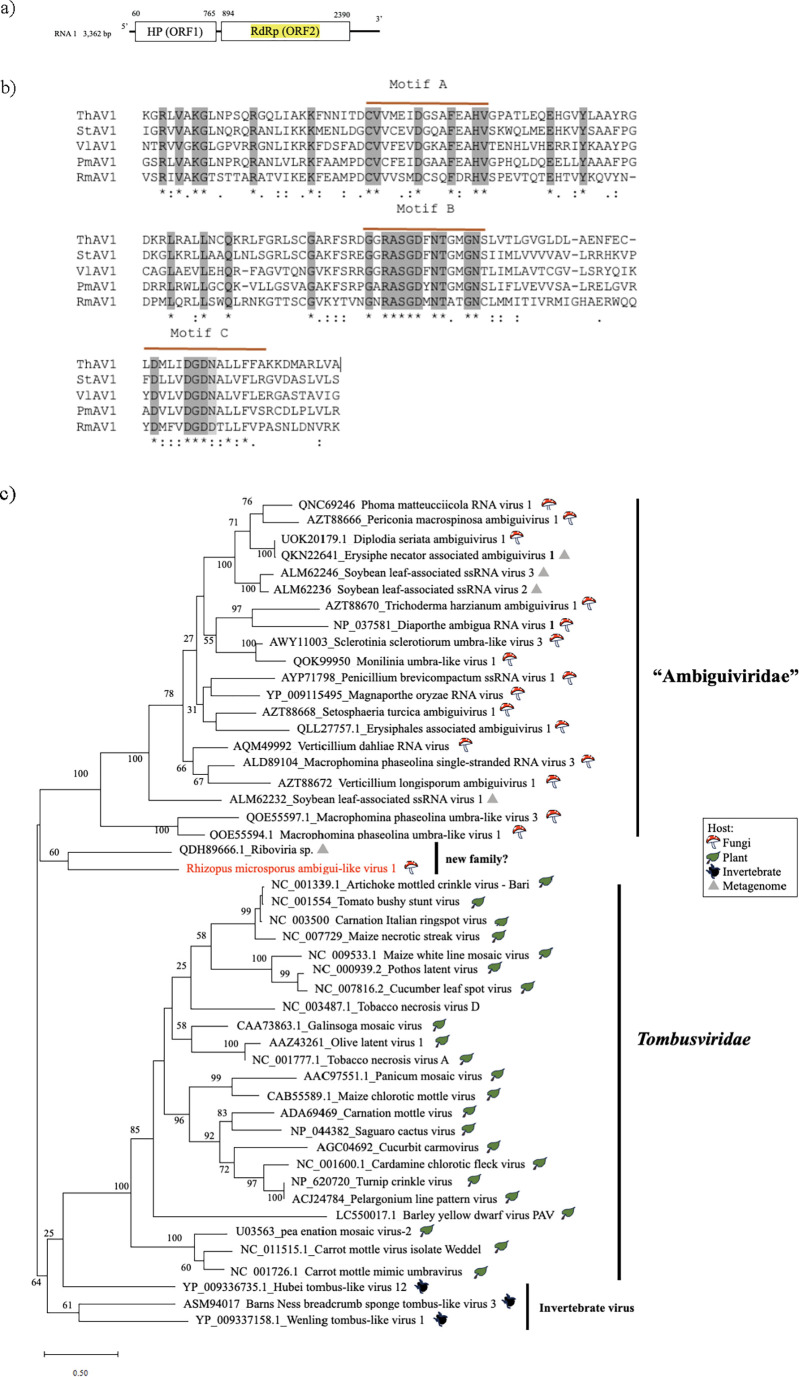
Characterization of RmAV1. (**a**) Genome organization of RmAV1; predicted ORFs are indicated by the white box. Hp, hypothetical protein. (**b**) Multiple amino acid sequence alignment of RdRPs encoded by RmAV1 and selected members of the proposed family “Ambiguiviridae.” Motifs A–C found in RdRP of +ssRNA viruses. ThAV1, *Trichoderma harzianum* ssRNA virus 1; StAV1, *Setosphaeria turcica* ssRNA virus 1; VlAV1, *Verticillium longisporum* ssRNA virus 1; PmAV1, Periconia macrospinosa ssRNA virus 1. (**c**) Phylogenetic tree of RdRPs of RmAV1, ambigui-like viruses, and tombusvirids (plant viruses) computed with RAxML and the PROTGAMMALG model, with 1,000 bootstrap replicates. Red font indicates mycoviruses found in this study. The scale bar shows the number of substitutions per site.

One of the *R. microsporus* mycoviruses shared a similarity with members of the proposed family “Ambiguiviridae” (40). This virus was detected in strain IFM 52934 and was designated *Rhizopus microsporus* ambigui-like virus 1 (RmAV1) (Table 1). Its complete genome is 3.3 kb, with two ORFs (ORF1 and ORF2) encoding a 241 aa hypothetical protein and a 498 aa putative RdRP, respectively (Fig. 6a). A multiple sequence alignment showed that RmAV1 RdRP contains the GDD triad in conserved motif C, instead of the GDN that occurs in other members of “Ambiguiviridae” (40). A BLASTp homology search with ORF1 and ORF2 showed the greatest similarity to *Riboviria* sp. (QDH89666) (36.61% and 42.23%, respectively). In a RdRP-based phylogenetic analysis, RmAV1 clustered with *Riboviria* sp. (QDH89666.1). The phylogenetic analysis revealed that RmAV1 clustered with *Riboviria* sp. with 62% support but was distinct from the proposed family “Ambiguiviridae,” suggesting the possible requirement for a new family (Fig. 6c).

### Novel +ssRNA virga-like virus from *R. microsporus*

**Fig 7:**
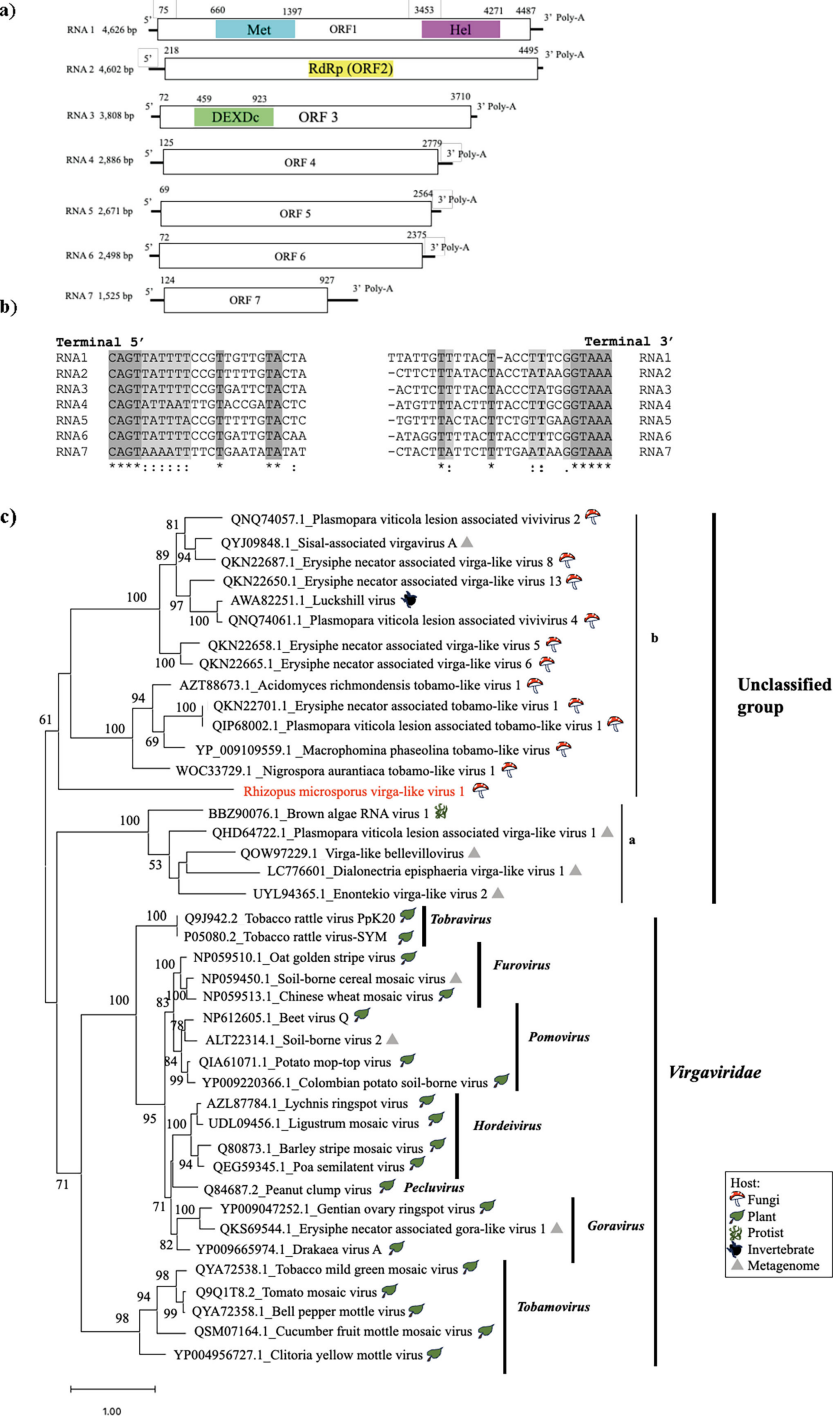
Characterization of RmVV1. (**a**) Genome organization of RmVV1; predicted ORFs are indicated by the white boxes, with the three conserved domains (Met, Hel, RdRP, and DEXDc). (**b**) Comparison of the 5′ and 3′ termini of RmVV1 segments (represented as DNA sequences). (**c**) Phylogenetic tree of RdRPs of RmVV1, related virga-like viruses, and virgavirids (plant viruses) computed with RAxML and the PROTGAMMALG model, with 1,000 bootstrap replicates. The red font indicates mycoviruses found in this study. The scale bar shows the number of substitutions per site.

Typical members of the family *Virgavridae* are nonsegmented or have +ssRNA genomes with a maximum of three segments (41). Here, we identified a seven-segment virga-like virus designated *Rhizopus microsporus* virga-like virus 1 (RmVV1). All segments had a conserved 5′ terminal sequence of four nucleotides (5′-CAGU…) and a conserved 3′ terminal sequence (…GU-3′) with a poly(A) tail (Fig. 7a and b). RmVV1 RNA1 encodes viral helicase superfamily 1 and methyltransferase, RNA2 encodes RdRP, RNA3 encodes DEAD-like helicase (DEXDc, smart00487), and the other segments encode hypothetical proteins. The lengths of the segments are 4.6, 4.5, 3.8, 2.8, 2.6, 2.4, and 1.5 kb, respectively (Table 1; Fig. 7a).

A BLAST search showed that the RNA1 protein shares similarity with that of *Bemisia tabaci* bromo-like virus 2 QWC36509.1 (24.88%) and that RNA2-encoded RdRP is similar to that of *Acidomyces richmondensis* tobamo-like virus 1 AZT88673.1 (26.39% identity). This RNA2 RdRP is also similar to nonviral hypothetical proteins encoded by shotgun sequences from the *R. arrhizu*s genome (KAG0754907.1, KAG0930863.1, KAG0973898.1, KAG0738144.1, KAG0822851.1, KAG0824072.1, KAG1060063.1, KAG0972937.1, KAG0734169.1, KAG0972793.1, and KAG1014630.1) with 30.11%–64.32% identity. The RNA3 protein shares the greatest similarity with its counterparts in *Aspergillus flavus* virga-like virus 1 (26.21%) (BED98334.1) and *Circinella minor* (22.82%) (KAG2223442.1). RNA4 and RNA5 share no similarity with any other sequence in a public database. RNA6 and RNA7 encode proteins similar to the hypothetical proteins of *R. arrhizus* (30.2%) and *Mucor plumbeus* (32.49%), respectively. We confirmed viral sequences of RNA7 by comparing the results of genomic PCR and nonquantitative RT-PCR (see Fig. S3). A phylogenetic analysis of RdRP suggested that RmVV1 belongs to an unclassified viral group related to the family *Virgaviridae*, which contains two major clades [(a) and (b) in Fig. 7c]. RmVV1 forms a clade with a long node that is related to clade (a) but distinct from the family *Virgaviridae* and clade (b) (Fig. 7c). The inclusion of a unique hepta-segmented virus in the clade of the unclassified group related to *Virgaviridae* leads to taxonomic ambiguity.

### Novel −ssRNA phasma-like virus from *R. microsporus*

**Fig 8:**
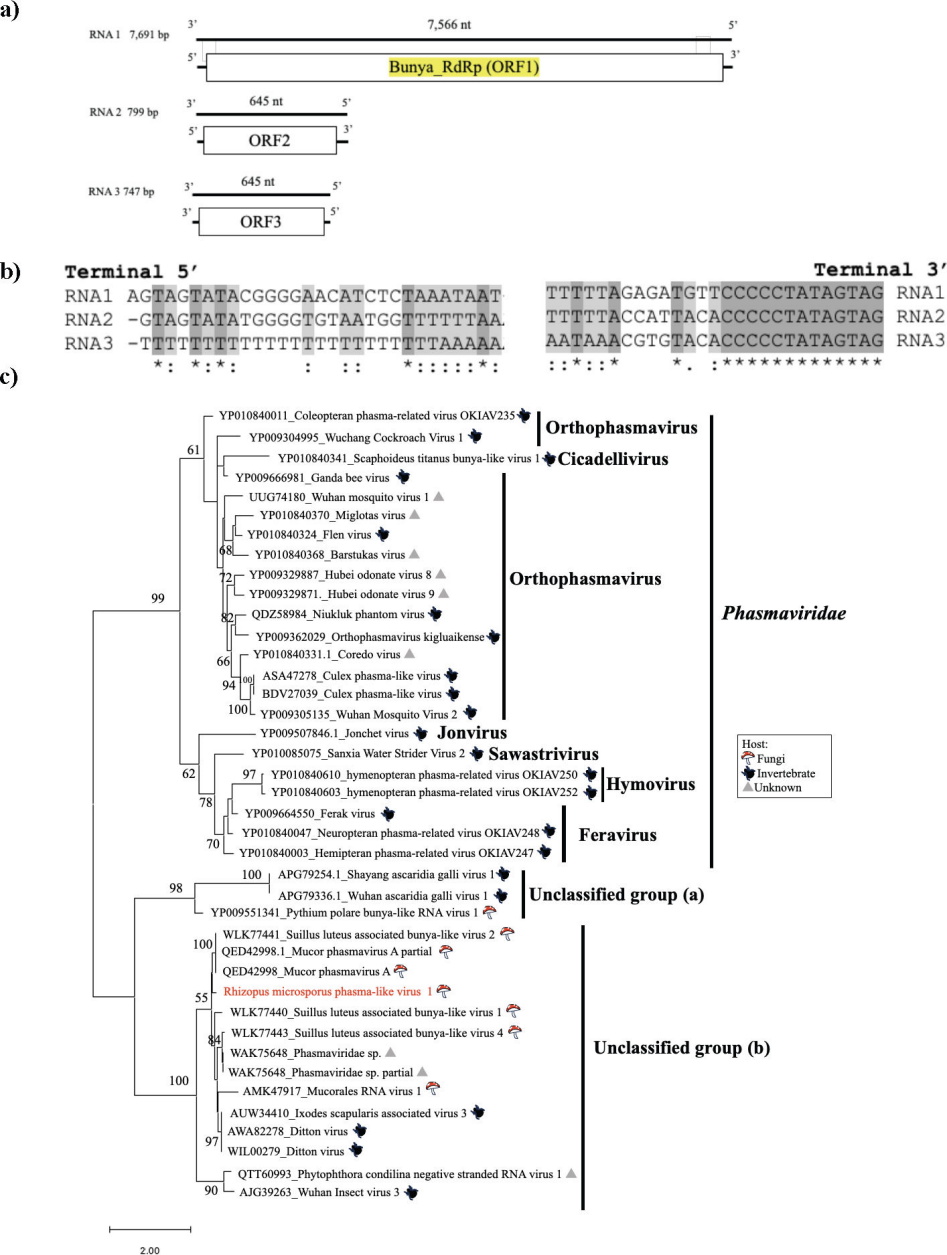
Characterization of RmPhV1. (**a**) Genome organization of RmPhV1; predicted ORFs are indicated by the white boxes. (**b**) Comparison of 5′ and 3′ termini of RmPhV1 genomic segments (represented as DNA sequences). (**c**) Phylogenetic tree of RdRPs of RmPhV1, phasma-like viruses, and phasmavirids computed with RAxML and the PROTGAMMALG model, with 1,000 bootstrap replicates. The red font indicates mycoviruses found in this study. The scale bar shows the number of substitutions per site.

Only one linear (−) ssRNA virus was detected in this study. −ssRNA viruses typically infect animals and plants (42, 43), but the study of Kondo et al. (44) reported the presence of a −ssRNA virus in fungi. The newly identified −ssRNA virus infecting *R. microsporus* was designated *Rhizopus microsporus* phasma-like virus 1 (RmPhV1) and had three segments carrying oppositely oriented ORFs. These segments are 7.6, 0.7, and 0.7 kb long, respectively (Fig. 8a). The genome organization showed that RNA1 (2,521 aa) encodes RdRP, whereas RNA2 (214 aa) and RNA 3 (214 aa) have no conserved sequences. RmPhV1 RdRP (L protein) shares similarity with that of *Suillus luteus*-associated bunya-like virus 2 (69.24%) (WLK77441.1); RmPhV1 M protein and RmPhV3 S protein share similarity with those of *Mucor lusitanicus* KAF1800631 (51.37%), *Streptomyces* sp. WP_221911531 (54.79%), and *Mortierella alpina* KAF9285847 (51.58%). Interestingly, this tri-segmented –ssRNA virus, RmPhV1, has no highly conserved sequence at the 5′-termini of RNA1–3 (RNA3 has poly-U at the 5′-terminus), whereas the sequence at the 3′-termini (…CCCCCUAUAGUAG-3′) is conserved (Fig. 8b). On a phylogenetic tree, this virus was placed in the unclassified group (b) related to the family *Phasmaviridae* and may potentially become a new genus or family (Fig. 8c).

## DISCUSSION

The research into mycoviruses infecting Mucoromycota has been sparse (45). In the last 3 years, six fungal species have been reported to collectively harbor 21 novel mycoviruses: 5 in *Mucor hiemalis* (46), 11 in *Umbelopsis* strains (47), 1 in *R. oryzae*, 3 in *R. microsporus* (13), and 1 in *Rhizopus stolonifer* (48). In the present study, we have greatly expanded the virome of *R. microsporus*. We have demonstrated the presence of diverse mycoviruses in Japanese clinical strains of *R. microsporus*, including one reported in a previous study: 11 +ssRNA viruses (4 mitoviruses, 4 narna-like viruses, an endornavirus, an ambigui-like virus, and a virga-like virus) and 1 −ssRNA virus (a phasma-like virus). Therefore, this study reports the first −ssRNA virus detected in *R. microsporus*.

The FLDS method was introduced in 2016 and has been successfully used to efficiently detect the complete genomes of many types of RNA viruses (+ssRNA, −ssRNA, and dsRNA genomes) (12, 24, 27–32). Using this method, we successfully acquired the complete genomes of 12 mycoviruses, including those with multipartite genomes, in which even the coding sequences of the segments show no similarity to other known proteins. Importantly, a unique hepta-segmented virus (RmVV1) was successfully identified using a feature of the FLDS technology that allows the typing of multi-RNA segments within a virus based on terminal sequences.

The results collected in the present study show that several viruses (or their variants) in the families *Mitoviridae* and *Narnaviridae* are present in clinical populations of *R. microsporus*. Multiple mitoviruses or narna-like viruses were found in a single strain of *R. microsporus* (RmMV1, RmMV3, and RmMV4 in IFM 59234, and RmNV1, RmNV2, and RmNV3 in IFM 56170) (see Table 1). Similar results have also been reported in previous studies, in which variants of mitoviruses and a narna-like virus were detected in a single fungal strain, e.g., *Botrytis cinerea*, *Plasmopora viticola, Rhizoctonia solani*, and *Gremmeniella abietina* (9, 49–51). Three variants of RmMV2 were detected in strains IFM 56170, IFM 56177, and IFM 61043. Notably, strains IFM 56170 and IFM 56177 were isolated from the skin and endometrium of the same pregnant heifers, respectively. The ITS and LSU sequences suggest that the *R. microsporus* strains were derived initially from identical strains infecting the heifer and then spread to different parts of the heifer’s body. Therefore, the variants of RmMV2 in these fungal strains could also be derived from the same lineage. The coordinated diversification of a fungal host and mycovirus within a close lineage is not well understood and is an issue for future study.

The family *Endornaviridae* consists of two genera, *Alphaendornavirus* and *Betaendornavirus* (38), and recently, the genus “*Gammaendornavirus*” was proposed by Luo et al. (39). All three known *R. microsporus* endornaviruses are related to the alphaendornaviruses, whereas only RmEV3 has a shorter genome than typical endornaviruses (see Fig. 5a). Similarly, RmEV3 was distantly related to other alphaendornaviruses in a phylogenetic analysis (Fig. 5b) and thus may be considered a member of a new genus, although it may also be a new species in the genus *Alphaendornavirus*.

Members of “Ambiguiviridae” encode two ORFs in a nonsegmented +ssRNA genome of 2.6–4.5 kb. The UAG stop codon is located in ORF1, and the second ORF (ORF2) is probably expressed as a fusion protein after a translational read-through of the ORF1 stop codon, similar to the replicates of plant viruses in the family *Tombusviridae* (40) (Fig. 6). RmAV1 may have a similar genome architecture, but its RdRP has a GDD triad in motif C instead of the GDN of other ambigui-like viral RdRPs (40). A phylogenetic analysis revealed a distant relationship between RmAV1 and the ambigui-like viruses, similar to some invertebrate viruses. The new clade that accommodates RmAV1 and *Riboviria* sp. (QDH89666, from a grassland soil sample) may be filled with other as-yet-undetected related viruses in the future and will possibly constitute a new family.

RmVV1 and many other relatives from fungi are distinct from plant-infecting members of the family *Virgaviridae*, in generally having a tri-segmented or nonsegmented RNA genome, whereas the majority of plant virgavirids have mono- or bi-segmented (+)RNA genomes (41). These fungus-infecting virgavirids may be classified as a new family (Fig. 7). Interestingly, the top 13 BLAST hits for RmVV1 RdRP included hypothetical proteins from *R. arrhizus* (52) and a hypothetical protein from *Eutypa lata* (KAI1255280, Ascomycota). RNA6 and RNA7 events (see Results) are still difficult to discuss in the context of horizontal gene transfer (HGT), but it is possible that the virus acquired them in the same way that the phasma-like virus segments were acquired, as discussed below. Interactions between hosts and non-retro-RNA viruses can accidentally cause HGT in the genomes of eukaryotic organisms, including plants, insects, and fungi (43, 44, 53). Similar events may also have occurred between the RmVV1 ancestor(s) and its fungal host(s), allowing the endogenization of viral sequences. The mechanism underlying the endogenization of viral sequences and the significance of such virus-derived sequences are still not well understood. However, it is an interesting event and represents one aspect of long-term virus–host interactions.

Although −ssRNA viruses are usually associated with animals and plants, some −ssRNA viruses have recently been discovered in fungi (54–56), after their first report in 2013 (44). Members of the family *Phasmaviridae* (order *Bunyavirales*) have a tri-segmented −ssRNA genome and are typically found in invertebrates (57). Recently, several phasma-like viruses have been reported in fungal hosts, such as *S. luteus* and *Mucor* spp. (58, 59). In the present study, RmPhV1 formed a well-supported sister clade to *Phasmaviridae*, together with viruses of *S. luteus* and *Mucor* spp. (see Fig. 8). However, of the viruses detected, only RmPhV1 has the additional small segments that are typically found in the classical members of the order *Bunyavirales* (60). These small putative RmPhV1 segments encode proteins of unknown function but show significant homology to genes thought to be of the host or possibly of endogenous bacterial origin. Their presence in RmPhV1 may have resulted from recombination between a viral genome and a putative viral–host chimeric mRNA, potentially generated by cap-snapping (61). However, further verification is required.

Earlier studies of mycoviruses detected in *R. microsporus* reported fewer viral taxa than our study, in which we detected more diverse mycoviruses and new taxa by adapting the FLDS method. However, only 6 of the 25 fungal strains tested were observed to harbor RNA viruses, including +ssRNA and −ssRNA viruses, but not dsRNA viruses, DNA viruses, or circular RNA viruses. The infection of fungi by dsRNA viruses has not been reported, although this type of virus is known to be omnipresent in fungi (62, 63). Furthermore, the fungal hosts tested were clinical strains collected from human and animal tissues, including skin, bronchoalveolar lavage fluid, leg, intestinal tract, and sputum (Table S1). Therefore, all these fungal strains may be related to disease manifestations in human or animal hosts. Therefore, it is reasonable to assume that clinical fungal strains are subject to selection pressures and are, therefore, biased toward the presence of viruses. This assumption is supported by previous studies that screened clinical strains of *R. microsporus*, largely from skin and sputum, for mycoviruses and found only +ssRNA viruses belonging to the families *Mitoviridae* and *Endornaviridae* (13). If dsRNA viruses reduce the virulence of *R. microsporus* in human and animal hosts, it may be difficult to detect them in clinical isolates. Further screening of non-biased *R. microsporus* collections for viruses could identify viruses associated with *R. microsporus* that are potential therapeutic agents for use against this fungus.

## MATERIALS AND METHODS

### Fungal strains

In total, 25 clinical isolates of *R. microsporus* were obtained from the Medical Mycology Research Center, Chiba University, Japan. They were collected through the National Bio-Resource Project, Japan. These strains were cultured in potato dextrose broth for 5–6 days at 30°C. The strains were identified using a primer set (ITS5 and ITS4) that amplified the ITS region (64) and a primer set (NL1 and NL4) that amplified the large subunit (LSU) rRNA gene region (65). All the fungal strains were stored in 10% glycerol at −80°C.

### RNA extraction, dsRNA fragmentation, and dsRNA ligation with an oligo adapter

Using a sterilized mortar and pestle, the total nucleic acids were extracted from about 0.2 g of frozen fungus in liquid nitrogen. The extraction process was continued with the addition of phenol/chloroform/isoamyl alcohol (ratio 25:24:1) at pH 5.2 and 500 µL of dsRNA extraction buffer, containing 20 mM Tris–HCl (pH 6.8), 200 mM NaCl, 2 mM EDTA, 1% SDS, and 0.1% (vol/vol) β-mercaptoethanol. The dsRNA was purified with a Micro Bio-Spin Chromatography Column (empty Bio-spin column, Bio-Rad, Hercules, CA, USA) containing cellulose powder (Cellulose D; Advantec, Tokyo, Japan), as described previously (28). It was then washed with 1× STE buffer containing 100 mM NaCl, 1 mM EDTA, 10 mM Tris/HCl (pH 8.0), and 16% ethanol (EtOH). The washed dsRNA was eluted with 300 µL of 1× STE. The final product was separated into two tubes: (i) 50 µL was stored at −80°C for FLDS and (ii) 250 µL was used for detecting dsRNA with AGE. The 250 µL sample was precipitated by the addition of 25 µL of sodium acetate and 500 µL of EtOH and incubated at −20°C for 30 min. The product was dried, and 10 µL of 1× STE was added to resuspend the pellet. AGE was performed at 100 V for 85 min, and the gels were stained with GelRed (Biotium, Inc.) in 1× Tris–acetate–EDTA (TAE) buffer.

To eliminate the remaining DNA and ssRNA before the FLDS analysis, the eluted dsRNA fraction was treated with DNase I (Invitrogen) and S1 nuclease (Invitrogen) at 37°C for 2 h, as described previously (28). In brief, the purified dsRNA was fragmented with a Covaris S220 Focused-ultrasonicator (Woburn, MA, USA), and the U2 primer adapter was ligated to the 3′ end of the fragmented dsRNA with T4 RNA ligase (Takara Bio Inc., Kusatsu, Japan).

### cDNA synthesis, library construction, and sequencing

cDNA was synthesized from the U2-primer-ligated dsRNA with the SMARTer 5′/3′ Kit (Takara Bio) and the U2-comp primer (28). The cDNA was stored at −80°C or amplified immediately with PCR. After PCR amplification with the U2-comp primer and universal primer mix provided with the kit, the cDNA was fragmented with an ultrasonicator (Covaris S220). Libraries were constructed with the Kapa HyperPrep Kit (Kapa Biosystems) for the Illumina platform. The libraries were sequenced with the Illumina MiSeq Reagent Kit v3 on the Illumina MiSeq platform (Illumina, California, USA).

### Data processing

Clean reads (0.3–1.8M reads per single strain sample) were obtained by removing the adaptor, low-quality reads, rRNA, and low-complexity sequences, as described previously (66). The raw sequence data were deposited to DDBJ as DRR567811 to DRR567816. The resultant reads were assembled with *de novo* assembly using CLC Genomic Workbench ver. 11.0 (CLC Bio, Aarhus, Denmark). We mapped the CLC reads with parameter 0.5 similarity of fraction and 0.8 length of fraction. To obtain the complete genomes of the viral RNA, the termini of the contigs were extended with Tablet version 1.19.09.03, CLC Genomics Workbench, and Genetyx ver. 14 (Genetyx). When the termini of the contig had the same bases more than 10 reads or ended with polyA sequence (27), it is considered as full-length sequence. The segmented RNAs were identified according to the similarities of the segment termini. The collected contigs were identified with the BlastP program against the National Center for Biotechnology Information nonredundant database, with an *e*-value ≤1 × 10^−5^ and ≥80%.

### Phylogenetic analysis and RNA secondary structure prediction

Mycovirus ORFs were identified with the ORF finder program (https://www.ncbi.nlm.nih.gov/orffinder). The predicted amino acid sequences were aligned with MUSCLE (67) in MEGA 11 (68) using the default parameters. trimAl was used to remove alignment-ambiguous positions (69). A phylogenetic tree was constructed based on the RdRP domain with the maximum-likelihood method RAxML (70) and 1,000 bootstrap replicates. The amino acid substitution model was selected with Aminosan (71) and assessed with the corrected Akaike information criterion (72). FigTree v.1.4.4 (73) and MEGA were used to visualize the phylogenetic tree. We used the RNAfold Web Server (http://rna.tbi.univie.ac.at//cgi-bin/RNAWebSuite/RNAfold.cgi) to predict the RNA secondary structures of the untranslated regions in the mycoviral genomes. Additionally, Bayesian analysis was performed using MrBayes to confirm similarity with the maximum-likelihood method (Fig. S4).

### RT-PCR

*The R*. *microsporus* fungal strains were inoculated onto potato dextrose agar plates from the glycerol stocks. After 3 days in culture, a small number of mycelia were picked from each plate with a sterile toothpick and placed into a PCR tube containing the mixture from the Express one-step superscript qRT-PCR Kit Universal (Thermo Fisher Scientific, Milford, CT, USA), in three replicates. With this method, we confirmed the presence of mycovirus(es) (6). The PCR product was separated in agarose gel stained with GelRed (Biotium, Hayward, CA, USA) in 1× TAE buffer for 28 min at 100 V. The primer sets used are listed in Table S2.

To test if the sequences are originated from genomic DNA, genomic PCR was performed using KOD one according to the product instruction (Toyobo, Osaka, Japan).

## SUPPLEMENTAL MATERIAL

Supplemental material msphere.00345-24-s0001.pdf Tables S1 and S2 and Figures S1 to S4.

ASM does not own the copyrights to Supplemental Material that may be linked to, or accessed through, an article. The authors have granted ASM a non-exclusive, world-wide license to publish the Supplemental Material files. Please contact the corresponding author directly for reuse.
