# Supplementary material for: New lineages of RNA viruses from clinical isolates of Rhizopus microsporus revealed by fragmented and primer-ligated dsRNA sequencing (FLDS) analysis
Source: mSphere. 2024 Jul 29;9(8):e00345-24. doi: 10.1128/msphere.00345-24 (PMC11351042; doi:10.1128/msphere.00345-24)

Supplementary Table S1. The fungal host information and the virus-infected strain

**Strain (IFM)**	**Fungal name**	**Source**	**Isolated date**	**Accession number (ITS)**	**dsRNA (AGE)**	**FLDS**
46008	*Rhizopus microsporus*	Allergic bronchopulmonarymycosis		LC390248		
47757	*Rhizopus microsporus*	Sputum	1998	LC390251		
51943	*Rhizopus microsporus*	trachea secretion	2002/08/30	LC390254		
**52934**	*Rhizopus microsporus*	Leg (horse) - dermatophytosis	2003	LC390255	√	√
**56170**	*Rhizopus microsporus*	Skin (pregnant heifer)- endometriosis	2007/10/04	LC390257	√	√
**56177**	*Rhizopus microsporus*	Endometrium (pregnant heifer)- endometriosis	2007/10/04	LC818998	√	√
57674	*Rhizopus microsporus*	Pus near left cervical lymph node and thyroid gland	2009/02	LC818999		
58027	*Rhizopus microsporus*	Sputum	2009	LC390258		
59626	*Rhizopus microsporus*	Swelling fluid	2010/09/03	LC390267		
59707	*Rhizopus microsporus*	Intraperitoneal necrotic tissue	2010/11	LC390269		
59833	*Rhizopus microsporus*	Lung	2010	LC390270		
60828	*Rhizopus microsporus*	Intrabronchially specimen	2011/10	LC390271		
60957	*Rhizopus microsporus*	Lung	2011	LC390272		
**61043**	*Rhizopus microsporus*	Intestinal tract	2011/12	LC390273	√	√
61110	*Rhizopus microsporus*	Lung	2011	LC390274		
61470	*Rhizopus microsporus*	Blood	2012	LC819000		
61906	*Rhizopus microsporus*	BALF	2013/02	LC390276		
62068	*Rhizopus microsporus*	Pus (lung)	2013/04	LC390277		
**62248**	*Rhizopus microsporus*	BALF	2012	LC390278	√	√
63585	*Rhizopus microsporus*	Blood	2015	LC390279		
63684	*Rhizopus microsporus*	Lung	2015	LC390280		
63708	*Rhizopus microsporus*	Skin	2015	LC819001		
**65465**	*Rhizopus microsporus*	Sputum	2018	LC819002		√
65629	*Rhizopus microsporus*	Feces	2018	LC819003		
65772	*Rhizopus microsporus*	Sputum	2018	LC819004		

***Yellow highlight indicates the fungal host carrying RNA viruses**

Supplementary Table S2. The list of primers for detecting mycoviruses in this study by RT-PCR

Host (IFM)	Viral name (abbreviation)	Forward	Reverse	Size (bp)
52934	Rhizopus microsporus mitovirus 1 (RmMV1)	CATTACGCTAGGAGGGCCTG	TGATATTCCTCAGCGACGGC	624
	Rhizopus microsporus mitovirus 3 (RmMV3)	AGTCCGCCTCCAGGGCATAGC	TCTCTACTAACCCGTGTAAAGGG	487
	Rhizopus microsporus mitovirus 4 (RmMV4)	GCTCGATGGGCGATTCCTTA	CCCTTTTGGGAGCACAGCTA	899
	Rhizopus microsporus narnavirus 4 (RmNV4)	CTAAGGCGATCCCAATTGCG	TCCAAGCCGCAACAAGGTCA	507
	Rhizopus microsporus endornavirus 3 (RmEV3)	ACAACCAGCGGACTTGAGAG	TATTCTACGGGCTTCGTCGC	460
	Rhizopus microsporus ambigui-like virus 1 (RmAV1)	GTGCCCAAGCTGAAACAAGG	AGTGTTCATGTCGCCAGAGG	834
56170	Rhizopus microsporus narnavirus 1 (RmNV1)	AGGCAATCGTCAAGGGTCTG	CGAGGTTATAGGGCACGGAC	558
	Rhizopus microsporus narnavirus 2 (RmNV2)	CTCTCAAGCCTCGCTTCCAA	GGCATAGTCCTTTGGGCCAT	503
	Rhizopus microsporus narnavirus 3 (RmNV3)	GGAAAGAGGCTGGAAGGTCC	GCCTGATGGACCAGACACAA	405
	Rhizopus microsporus mitovirus 2 (RmMV2)	AGTCCGCCTCCAGGGCATAGC	TCTCTACTAACCCGTGTAAAGGG	487
	Rhizopus microsporus virga-like virus 1 (RmVV1)	GTACCTCGGGAGACACTTGC	AGCTAACAACTCCGAACCCG	516
		CGTCAGACCGTATTGGAAGT	GGTAGCAGTGACGAACGTTA	566
		TTCTATGTCGGAGGCGAAGC	TGACGCCAGGAACCTCATTC	452
		AGCGTAGATCGTCCCTTCCT	GCAACGAAAAGAGCAAGCCA	533
		ACCCAGTGTATCCGGTAATG	CTAGTCTGCGAAGCTACAAC	809
		TTGTTGCAGAATCGTGTGCG	TAGGCGCCATAGCGACAAAT	597
		ATTCTCGCGTAGGTAAGGAG	TTCCAGCGACCTCAAAGAGT	424
56177	Rhizopus microsporus mitovirus 2 (RmMV2)	AGTCCGCCTCCAGGGCATAGC	TCTCTACTAACCCGTGTAAAGGG	487
61043	Rhizopus microsporus mitovirus 2 (RmMV2)	AGTCCGCCTCCAGGGCATAGC	TCTCTACTAACCCGTGTAAAGGG	487
62248	Rhizopus microsporus mitovirus 3 (RmMV3)	AGTCCGCCTCCAGGGCATAGC	TCTCTACTAACCCGTGTAAAGGG	487
65465	Rhizopus microsporus phasma-like virus 1 (RmPhV1)	ACGTGACATGGCAGCATCAA	TGATTTCCGTGCCTGGTGAT	725
		GCCATTTGAGCCTGTTGGAA	TGACGTCAACAGAGGGAGATA	325
		CGATCTCCAGTTTCTTGCCCT	CACCAAGGCGACTTCAAAGA	308

Supplementary Figure S1. The full version of the phylogenetic tree of *Narnaviridae*

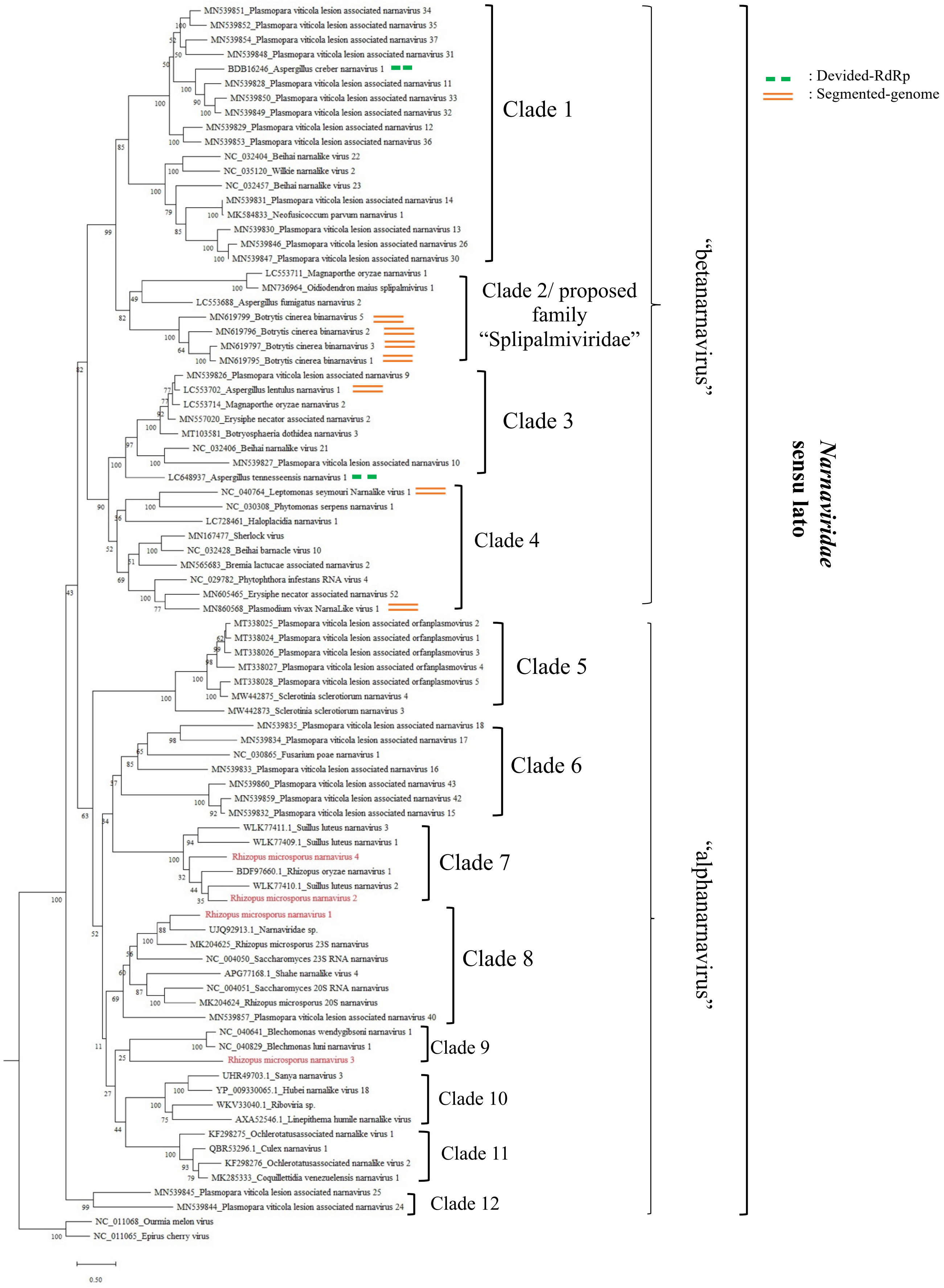

Supplementary Figure S2. The secondary structure of mycoviruses in this study

a) Rhizopus microsporus mitovirus 1 (RmMV1)

5’ terminal 3’ terminal

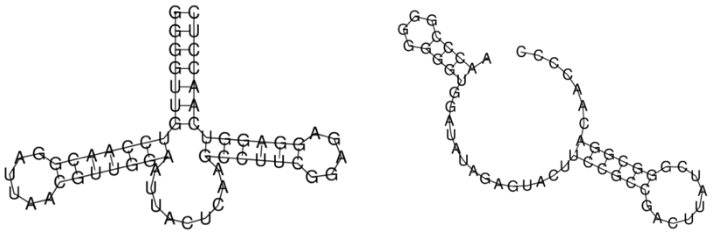

Energy: -24.40 kcal/mol Energy: -17.20 kcal/mol

b) Rhizopus microsporus mitovirus 2 (RmMV2)

5’ terminal 3’terminal

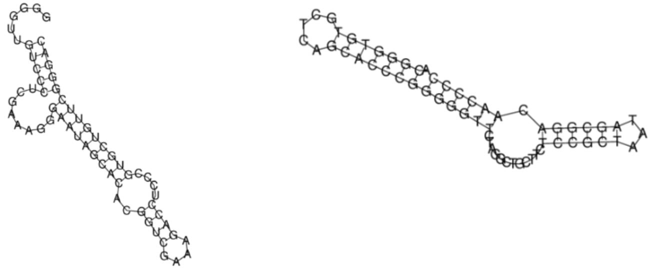

Energy: -23.40 kcal/mol Energy: -26.10 kcal/mol

c) Rhizopus microsporus mitovirus 3 (RmMV3)

5’ terminal 3’terminal

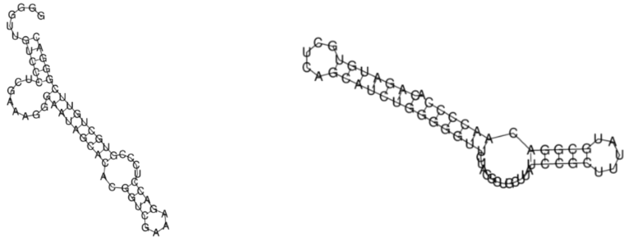

Energy: -23.40 kcal/mol Energy: -22.80 kcal/mol

d) Rhizopus microsporus mitovirus 4 (RmMV4)

5’ terminal 3’terminal

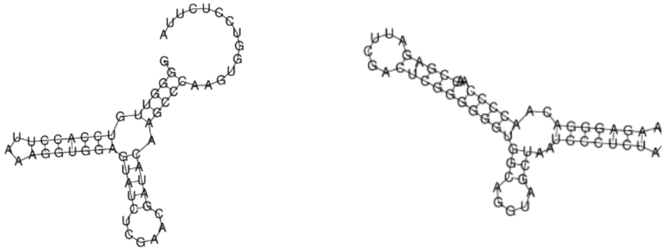

Energy: -19.40 kcal/mol Energy: -25.00 kcal/mol

e) Rhizopus microsporus narnavirus 1 (RmNV1)

5’ terminal 3’terminal

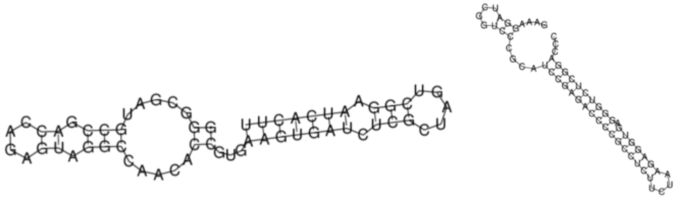

Energy: -14.10 kcal/mol Energy: -33.00 kcal/mol

f) Rhizopus microsporus narnavirus 2 (RmNV2)

5’ terminal 3’terminal

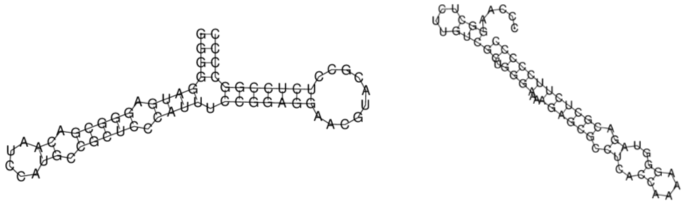

Energy: -24.20 kcal/mol Energy: -23.00 kcal/mol

g) Rhizopus microsporus narnavirus 3 (RmNV3)
5’ terminal 3’terminal

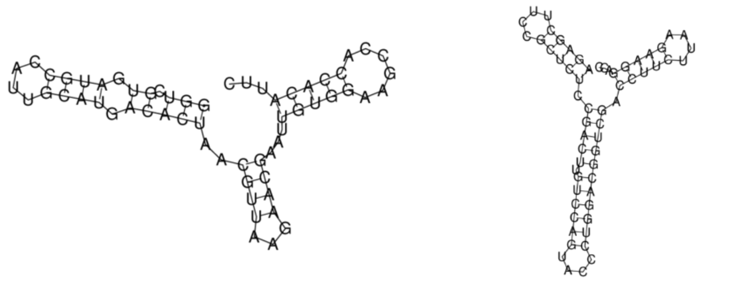

Energy: -13.90 kcal/mol Energy: -26.30 kcal/mol

h) Rhizopus microsporus narnavirus 4 (RmNV4)
5’ terminal 3’terminal

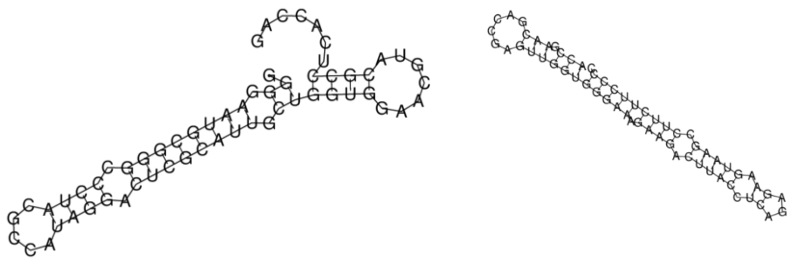

Energy: -22.80 kcal/mol Energy: -20.20 kcal/mol

i) Rhizopus microsporus endornavirus 3 (RmEV3)
5’ terminal 3’terminal

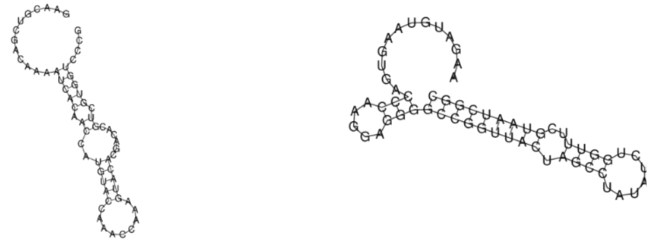

Energy: -5.40 kcal/mol Energy: -20.70 kcal/mol

j) Rhizopus microsporus ambigui-like virus 1 (RmAV1)
5’ terminal 3’terminal

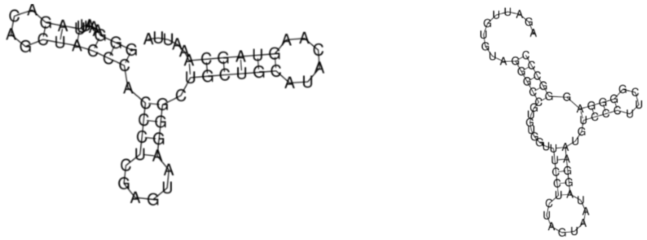

Energy: -15.20 kcal/mol Energy: -17.60 kcal/mol

k) Rhizopus microsporus virga-like virus 1 (RmVV1) -RNA1
5’ terminal 3’terminal

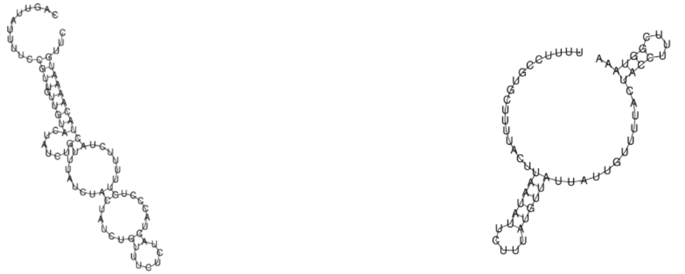

Energy: -0.60 kcal/mol Energy: -4.30 kcal/mol

l) Rhizopus microsporus virga-like virus 1 (RmVV1) -RNA2
5’ terminal 3’terminal

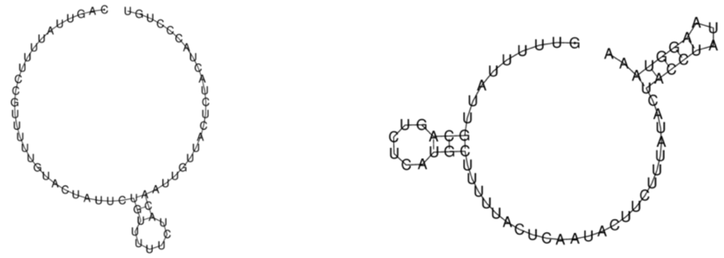

Energy: -0.30 kcal/mol Energy: -6.00 kcal/mol

m) Rhizopus microsporus virga-like virus 1 (RmVV1) -RNA3

5' terminal

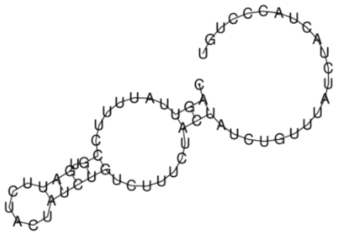

Energy: -0.30 kcal/mol

3'terminal

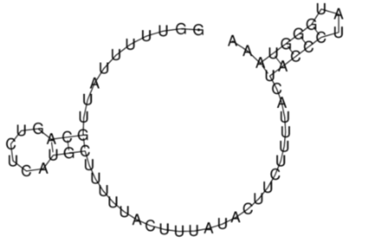

Energy: -7.70 kcal/mol

n) Rhizopus microsporus virga-like virus 1 (RmVV1) -RNA4

5' terminal

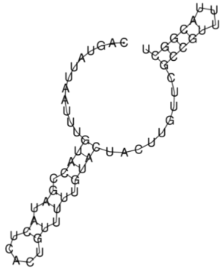

Energy: -10.90 kcal/mol

3'terminal

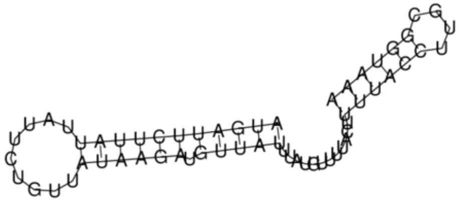

Energy: -7.60 kcal/mol

n) Rhizopus microsporus virga-like virus 1 (RmVV1) -RNA5

5' terminal

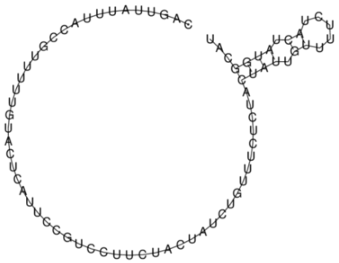

Energy: -10.20 kcal/mol

3'terminal

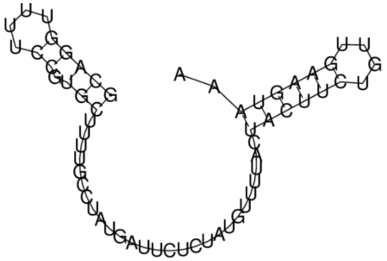

Energy: -7.60 kcal/mol

o) Rhizopus microsporus virga-like virus 1 (RmVV1) -RNA6

5' terminal

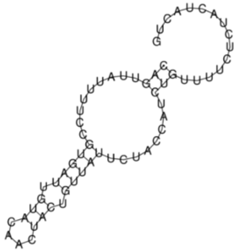

Energy: -3.10 kcal/mol

3'terminal

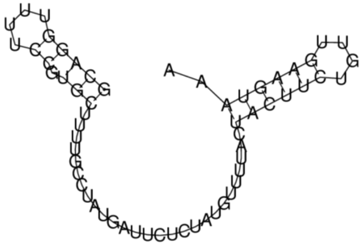

Energy: -8.90 kcal/mol

p) Rhizopus microsporus virga-like virus 1 (RmVV1) -RNA7

5' terminal

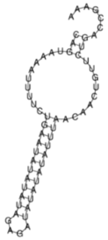

Energy: -9.80 kcal/mol

3'terminal

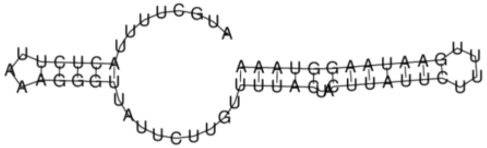

Energy: -9.40 kcal/mol

q) Rhizopus microsporus phasma-like virus 1 (RmPhV1) -RNA1

5' terminal

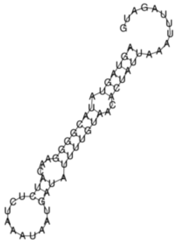

Energy: -5.90 kcal/mol

3'terminal

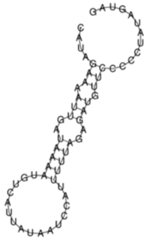

Energy: -2.80 kcal/mol

r) Rhizopus microsporus phasma-like virus 1 (RmPhV1) -RNA2

5’ terminal

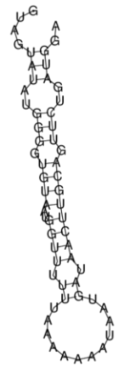

Energy: -5.90 kcal/mol

3’terminal

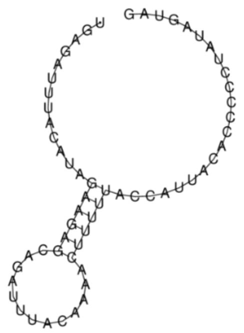

Energy: -1.00 kcal/mol

s) Rhizopus microsporus phasma-like virus 1 (RmPhV1) -RNA3

5’ terminal

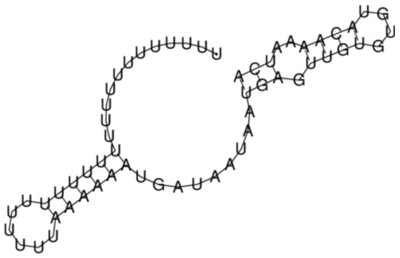

Energy: -1.90 kcal/mol

3’terminal

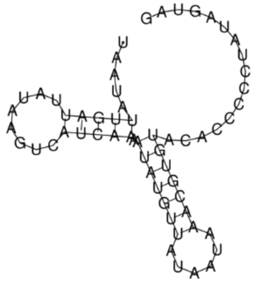

Energy: -5.30 kcal/mol

Supplementary Figure S3. The confirmation of segment 7 of RmVV1 using total nucleic acid as a template

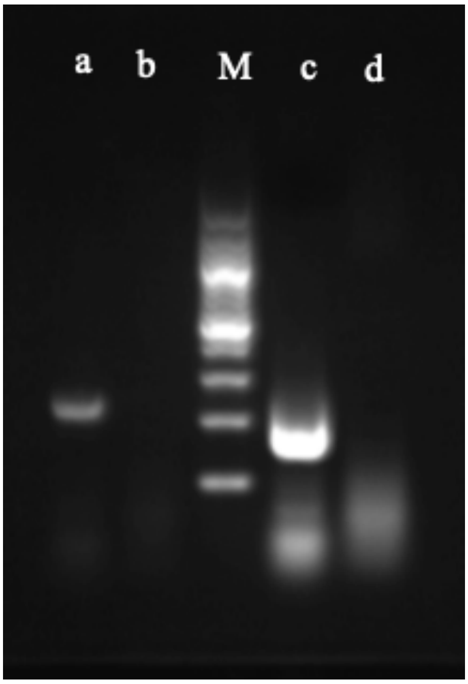

a: RT-PCR (1µl)
b: Genomic PCR, KOD one (1µl)
M: ladder 200 bp
c: RT-PCR (10µl)
d: Genomic PCR, KOD one (10µl)

Supplementary Figure S4. Bayesian phylogenetic analysis using MrBayes

Bayesian estimation ambigui-like virus

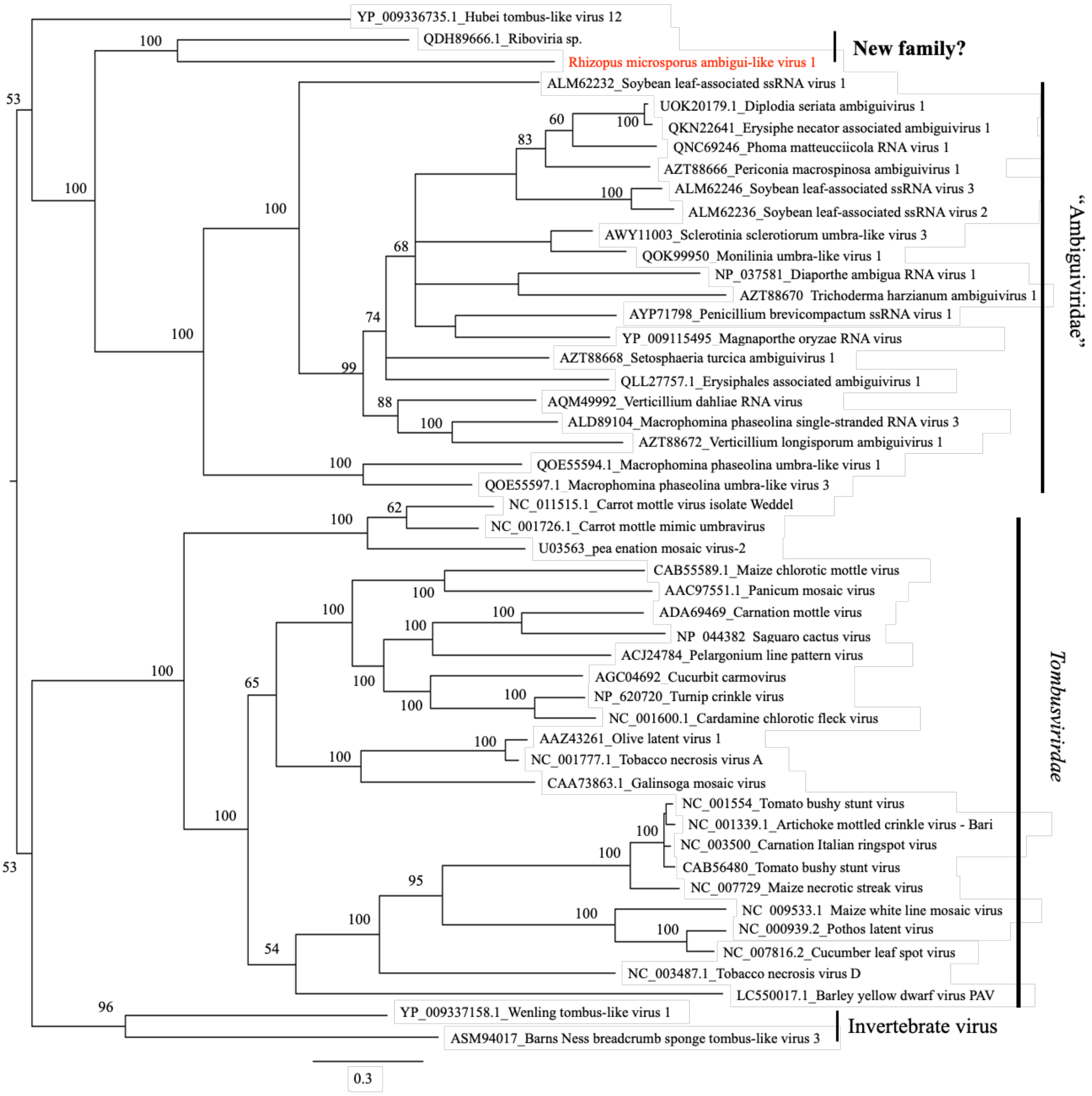

Bayesian estimation virga-like virus

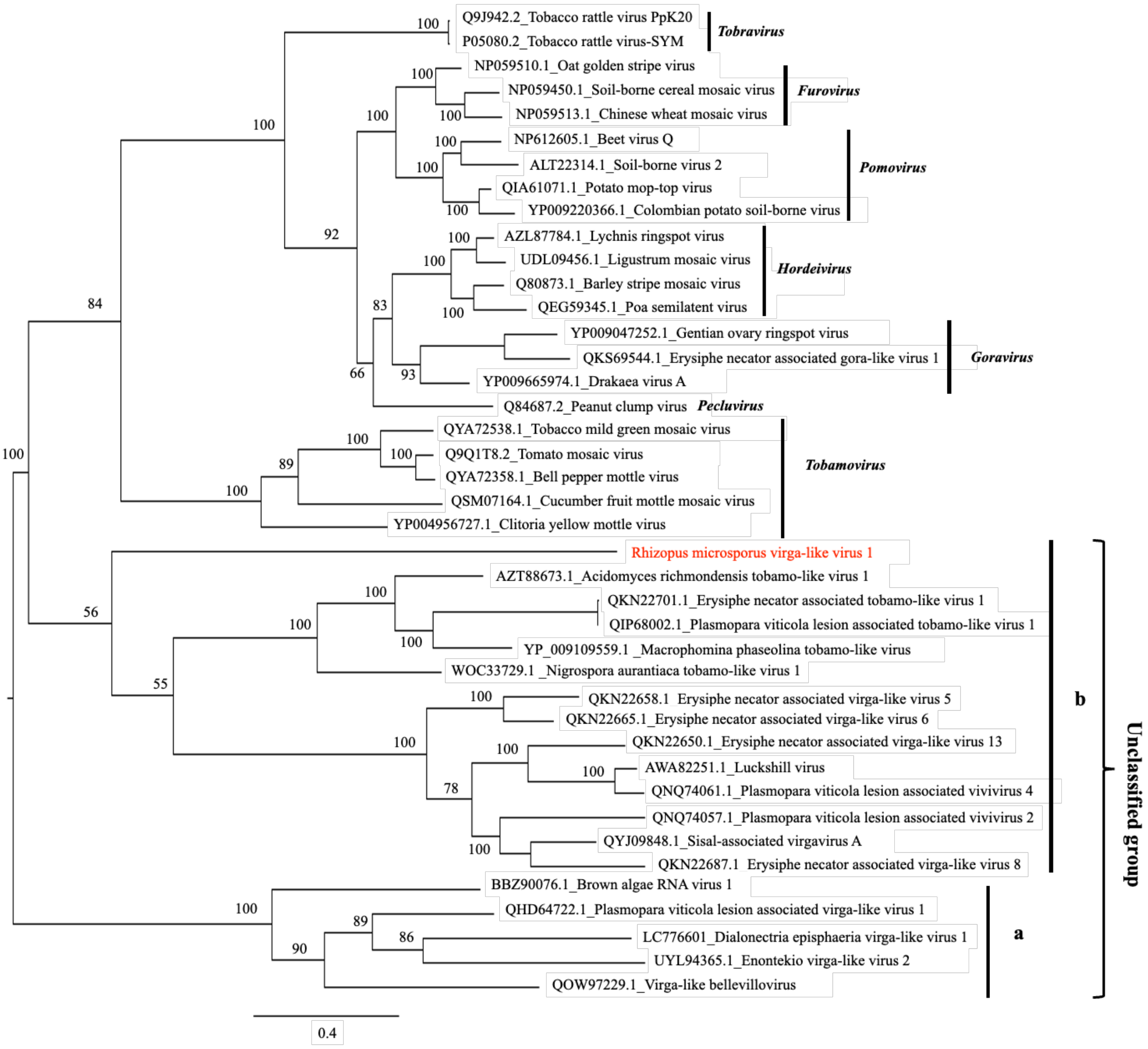

Bayesian estimation phasma-like virus

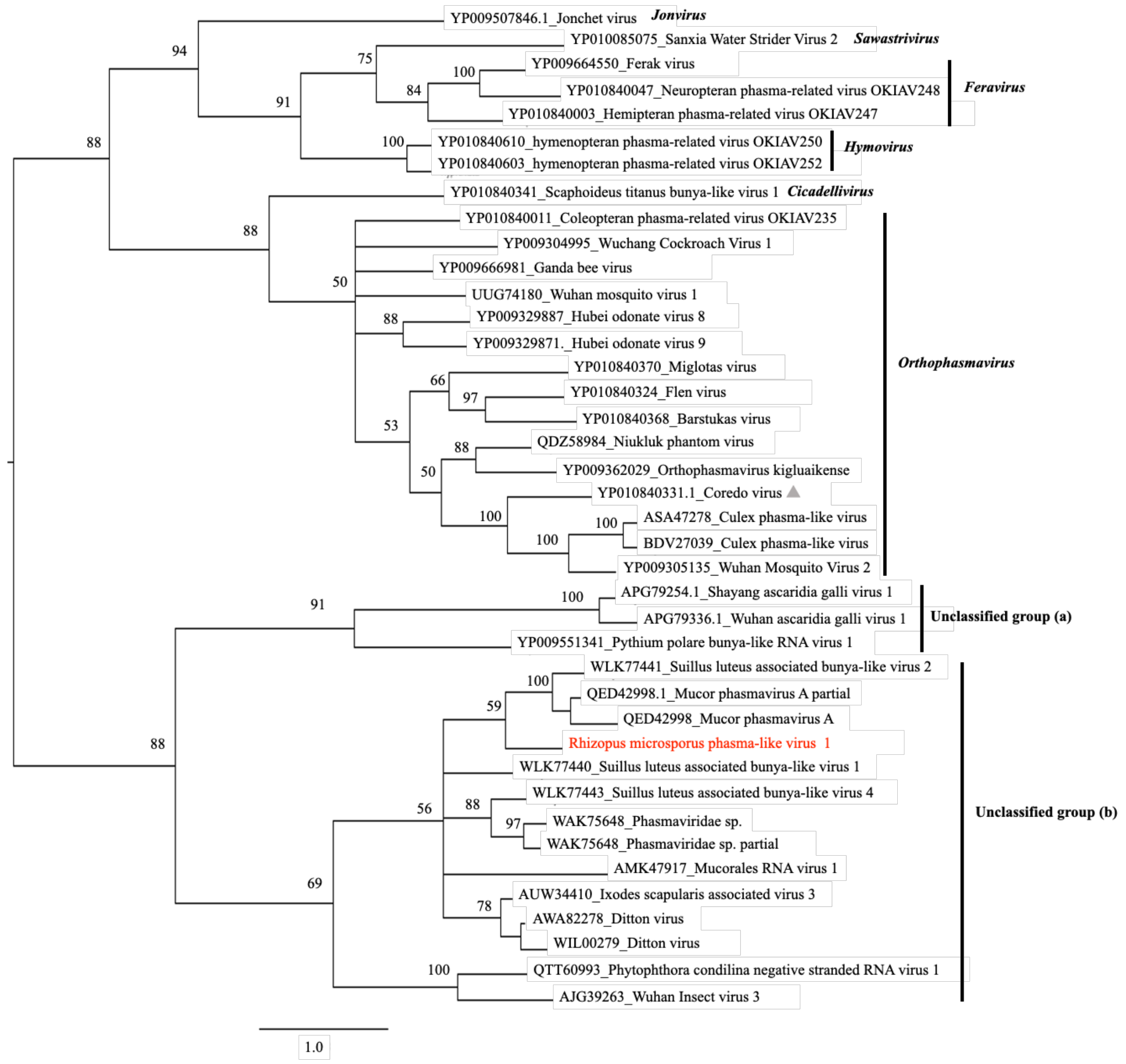

Supplement: Supplemental material — Tables S1 and S2 and Figures S1 to S4. [file msphere.00345-24-s0001.pdf]
